# Persistent Pain After Breast Cancer Treatment, an Underreported Burden for Breast Cancer Survivors

**DOI:** 10.1245/s10434-024-15682-2

**Published:** 2024-06-28

**Authors:** Bo T. M. Strijbos, Loes Janssen, Adri C. Voogd, Willem A. R. Zwaans, Rudi M. H. Roumen, Adriana J. G. Maaskant-Braat

**Affiliations:** 1 Department of Surgical Oncology, Máxima Medical Center, Veldhoven, The Netherlands; 2 Department of Epidemiology, Maastricht University, Maastricht, The Netherlands; 3 NUTRIM School of Nutrition and Translational Research in Metabolism, Maastricht University, Maastricht, The Netherlands

**Keywords:** Breast cancer, Breast cancer treatment, Pain, Postoperative, Persistent pain, Surgery breast

## Abstract

**Background:**

Many patients who have undergone surgery experience persistent pain after breast cancer treatment (PPBCT). These symptoms often remain unnoticed by treating physician(s), and the pathophysiology of PPBCT remains poorly understood. The purpose of this study was to determine prevalence of PPBCT and examine the association between PPBCT and various patient, tumor, and treatment characteristics.

**Patients and Methods:**

We conducted a questionnaire-based cross-sectional study enrolling patients with breast cancer treated at Máxima Medical Center between 2005 and 2016. PPBCT was defined as pain in the breast, anterior thorax, axilla, and/or medial upper arm that persists for at least 3 months after surgery. Tumor and treatment characteristics were derived from the Dutch Cancer Registry and electronic patient files.

**Results:**

Between February and March 2019, a questionnaire was sent to 2022 women, of whom 56.5% responded. Prevalence of PPBCT among the responders was 37.9%, with 50.8% reporting moderate to severe pain. Multivariable analyses showed that women with signs of anxiety, depression or a history of smoking had a higher risk of experiencing PPBCT. Women aged 70 years or older at diagnosis were significantly less likely to report PPBCT compared with younger women. No significant association was found between PPBCT and treatment characteristics, including type of axillary surgery and radiotherapy.

**Conclusions:**

A considerable percentage of patients with breast cancer experience PPBCT. Women with signs of anxiety or depression and women with a history of smoking are more likely to report PPBCT. Further research is required to understand the underlying etiology and to improve prevention and treatment strategies for PPBCT.

Breast cancer is the most common cancer among women. In 2021, there were approximately 18,000 women with newly diagnosed breast cancer in the Netherlands,^1,2^ and breast cancer is the most common cause of death for women aged 30–59 years.^3^ Women with a diagnosis of nonmetastatic early-stage breast cancer will, in general, undergo primary surgery of the breast, either lumpectomy or mastectomy, as well as a sentinel node biopsy procedure with or without radiation therapy. Depending on tumor characteristics, adjuvant systemic therapy consisting of endocrine therapy, chemotherapy and/or targeted therapy, may be offered. There is also the option for neo-adjuvant therapy.^4^ The 5-year relative survival rate for patients with breast cancer in the Netherlands has increased to approximately 88% as a result of earlier diagnosis owing to improved screening and treatment strategies.^2,3^ Due to this decrease in mortality rate, the focus has shifted toward improvement of physical function and quality of life by reducing the risk of treatment-related morbidity.^5,6^ In more recent years, clinical care of patients with breast cancer has further shifted toward a more patient-centered approach and more attention is given to patient-reported outcome measures (PROMs). PROMs are defined as feedback on a patient’s health condition, such as symptoms and quality of life, provided directly by the individual patient without external interpretation.^7^ This shift has also contributed to the increased focus on morbidity.^8^

A large proportion of patients surviving breast cancer who have undergone surgery experience persistent pain. Often this pain is localized in different regions such as the breast, axilla, upper arm, and the anterior and/or lateral chest wall.^14^ This has become an increasingly recognized condition that affects between 25 and 60% of these patients.^9–11^ The International Association for the Study of Pain (IASP) defines chronic post-surgical pain (CPSP) as pain that develops after a surgical procedure and persists beyond the normal healing process of 3 months.^12^ There are no universally accepted diagnostic criteria for persistent pain after breast cancer treatment (PPBCT). Currently, the criteria of the IASP for CPSP are used, unofficially, to define and diagnose PPBCT.^13^ PPBCT can predominantly be characterized as neuropathic pain.^9,14,15^ Several studies suggest that persistent neuropathic pain is triggered by peripheral nerve injury caused during surgical dissection or because of postoperative inflammation.^5,16^ Other studies suggest that chemotherapy and radiation therapy contribute to the development of persistent pain.^13,17,18^ Studies show that psychosocial factors, such as catastrophizing, anxiety, depression, somatization, and sleep quality, can play an important role in the development of PPBCT.^19,20^ However, the exact pathophysiology is poorly understood, which hampers the preoperative screening, counseling and management of patients experiencing PPBCT and also explains why PPBCT is often not recognized. Therefore, it is of clinical interest to identify potential risk factors that contribute to the reporting and development of PPBCT. Previous studies have reported risk factors for PPBCT, and these include axillary lymph node dissection,^9,10,22,23^ younger age,^9,10,23^ radiotherapy,^10,23^ acute postoperative pain,^10^ obesity (body mass index > 30),^23^ lower socio-economic status,^9^ endocrine treatment in post-menopausal woman,^22^ and non-Caucasian ethnicity.^9^ However, inconsistent results were found, with some studies showing negative associations and others reporting no associations at all.^24,25^ In particular, there is little agreement among studies in relation to the role of obesity in PPBCT.^25^

These contradictory results make it difficult to draw definitive conclusions concerning determinants of PPBCT. Accordingly, the purpose of this study was to examine the prevalence of PPBCT and the association between PPBCT and various patient, tumor, and treatment characteristics in women treated for breast cancer in a single institution in the Netherlands.

## Patients and Methods

### Study Eligibility Criteria

This study was designed as a retrospective questionnaire-based cohort study of patients who completed breast cancer surgery at Máxima Medical Center (Máxima MC), a large non-university teaching hospital in the Netherlands, between January 2005 and December 2016. Inclusion criteria were women aged 18 years or older at the time of diagnosis who had completed surgery for breast cancer more than 3 months prior to assessment. Patients with an inability to fill in the questionnaires due to a language barrier, patients who were already deceased, and patients with cognitive impairment were excluded from the study. Written informed consent was obtained from all participants.

### Data Collection

Patient data were retrieved from the population-based Eindhoven Cancer Registry. This registry records data from all newly diagnosed patients with cancer in the southeast region of the Netherlands, an area with approximately 2.4 million inhabitants. Eligible patients were identified and questionnaires were sent between February and March 2019.

The primary outcome was the prevalence of PPBCT. PPBCT was defined as pain located in the area of the breast, axilla, the lateral thorax, and/or the arm on the operated side of the body, persisting for more than 3 months after end of treatment. Patients were asked to fill out a questionnaire and were asked if they experienced PPBCT. Additionally, they rated their pain intensity on a zero to ten numerical rating scale (NRS) and a verbal rating scale (VRS). Pain was categorized as mild when the value on the NRS was less than or equal to 4. Values greater than 4 were categorized as moderate to severe pain. Questionnaires also included a portion of the McGill Pain Questionnaire Dutch Language Version (MPQ-DLV), questions from the Douleur Neuropathique (DN4), a section of the Multidimensional Pain Inventory Dutch Language Version (MPI-DLV), the Hospital Anxiety and Depression scale (HADS), and questions regarding pain management and substance use.

Different cutoff values were utilized in the questionnaires. In this study, participants completed a modified version of the DN4 questionnaire, which consisted of seven questions focusing solely on the presence of sensory symptoms associated with neuropathic pain. If a participant responded “Yes” to any of these questions, it was scored as positive for that particular symptom. The total score was then calculated by summing the positive responses. A score of 4 or higher was used as the cutoff value to classify the pain as neuropathic. The scoring system of the HADS consists of 14 items divided into two subscales: anxiety and depression. Each item is scored on a 4-point scale, with higher scores indicating higher levels of anxiety and depression. The total score for each subscale ranges from 0 to 21, with scores of 8 or higher considered clinically significant and indicative for the presence of anxiety or depression. Sent questionnaires contained a cover letter, two consent forms, and a stamped and addressed reply envelope. Patients were given the opportunity to fill out the questionnaire online, and a gentle reminder was sent 4 weeks later, if needed. Additional data on patient, tumor, and treatment characteristics were provided by the Dutch Cancer Registry, based on the pathology reports, and from the electronic patient records. Data were managed using Research Manager (data management software, Cloud9 software, Deventer).

### Statistical Analyses

The prevalence of PPBCT and various pain characteristics were assessed and presented as descriptive statistics, as appropriate. Patients were stratified into two categories: patients experiencing PPBCT and patients not experiencing PPBCT. These categories were based on the dichotomous (yes/no) outcome of the first question from the questionnaire, which was: “Do you currently have pain in the area of the chest, axilla, lateral side of the body, or arm on the side where you had breast cancer surgery?”. Patient, tumor, treatment, and pain characteristics were compared between groups using Pearson’s *χ*^2^ test or Fisher’s exact test, as appropriate. For categorical data an independent-samples *t*-test was used to compare continuous variables. Variables described in the literature as potentially associated with PPBCT were first assessed in a univariate logistic regression analysis. Variables that showed a significant association with PPBCT in the univariate analyses (*p*-value < 0.1) were entered in a multivariate analysis. Odds ratios (OR), 95% confidence intervals (95% CI), and corresponding* p*-values were calculated. A *p*-value of < 0.05 was considered statistically significant. Data analysis was done using SPSS® version 22 (IBM Corp, released 2020, Armonk, New York, USA).

### Ethical Approval

All procedures were conducted in accordance with institutional guidelines. The Ethics Committee of Máxima Medical Centre concluded that the rules laid down in the medical research involving human subjects act (also known by its Dutch abbreviation WMO) did not apply to this study (METC N18.151), and a formal ethical approval was waived.

## Results

### Response Rate

**Fig. 1:**
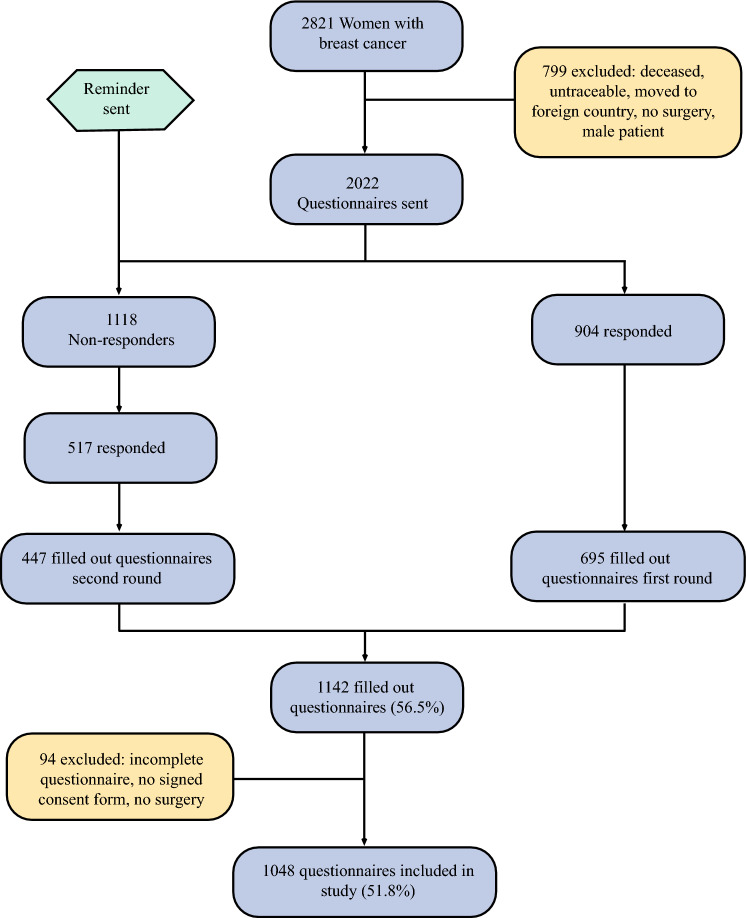
Flow-chart of questionnaire response and included patients

Questionnaires, including an informed consent form, were sent to 2022 eligible patients between February and March 2019. A total of 1142 patients completed the questionnaires (56.5% response rate). Ultimately, 1048 patients were included in this study (51.8%). Figure 1 contains a flow chart of the questionnaire response progress and the patients included in this study.

### Prevalence of PPBCT and Overall Characteristics

Overall, 397 (37.9%) patients reported PPBCT, and 651 (62.1%) patients reported no pain. Tables 1, 2 and 3 show the patient, tumor and treatment characteristics of the included patients.

**Table 1:** Patient characteristics

Patient characteristics	Number of patients (*n*)	No pain (*n* = 651, 62.1%)	Persistent pain (*n* = 397, 37.9%)	*p*-Value
*Age at diagnosis, years* (*SD*) (*n* = 1048, 100%)		59.53 (±11.525)	56.16 (±10.724)	< 0.001•
< 40	57	29 (4.5)	28 (7.1)	
40–50	213	118 (18.1)	95 (23.9)	
50–70	623	387 (59.4)	236 (59.4)	
≥ 70	155	117 (18.0)	38 (9.6)	
*BMI at baseline, kg/m*^2^ (*SD*) (*n* = 696, 66.4%)				0.322
< 18	8	4 (0.9)	4 (1.5)	
18–25	330	206 (48.1)	124 (46.3)	
25–30	232	145 (33.9)	87 (32.5)	
30–35	91	55 (12.9)	36 (13.4)	
35–40	20	13 (3.0)	7 (2.6)	
≥ 40	15	5 (1.2)	10 (3.7)	
Missing	352	223	129	
*Smoking* (*n* = 1035, 98.8%)				0.010•
Yes	93	55 (8.6)	38 (9.6)	
No	715	463 (72.3)	252 (63.8)	
Quit	227	122 (19.1)	105 (26.6)	
Missing	13	11	2	
*Drinking alcohol* (*n* = 1039, 99.1%)				0.380
Yes	604	380 (59.2)	224 (56.4)	
No	435	262 (40.8)	173 (43.6)	
Missing	9	9	0	
*Menopausal stage* (*n* = 654, 62.4%)				0.004•
Premenopausal	162	95 (23.2)	67 (27.5)	
Perimenopausal	59	27 (6.6)	32 (13.1)	
Postmenopausal	433	288 (70.2)	145 (59.4)	
Missing	394	241	153	
*Anxiety* (*n* = 1015, 96.9%)				0.000•
Yes (HADS ≥ 8)	200	83 (13.2)	117 (30.2)	
No (HADS < 8)	815	544 (86.8)	271 (69.8)	
Missing	33	24	9	
*Depression* (*n* = 1022, 97.5%)				0.000•
Yes (HADS ≥ 8)	121	44 (7.0)	77 (19.6)	
No (HADS < 8)	901	585 (93.0)	316 (80.4)	
Missing	26	22	4	

*SD* Standard deviation, *BMI* Body mass index. “•” indicates statistical significance. Note: variables as number (percentage)

**Table 2:** Tumor characteristics

Tumor characteristics	Number of patients (*n*)	No pain (*n* = 651, 62.1%)	Persistent pain (*n* = 397, 37.9%)	*p*-Value
*Breast cancer side* (*n* = 1048, 100%)				0.800
Left	540	331 (50.8)	209 (52.6)	
Right	487	306 (47.0)	181 (45.6)	
Double sided	21	14 (2.2)	7 (1.8)	
*Topography* (*n* = 1048, 100%)				0.741
Central part	43	26 (4.0)	17 (4.3)	
Medial upper quadrant	140	87 (13.4)	53 (13.4)	
Medial lower quadrant	56	32 (4.9)	24 (6.0)	
Lateral upper quadrant	373	231 (35.5)	142 (35.8)	
Lateral lower quadrant	89	57 (8.8)	32 (8.1)	
Mamma overlay	327	202 (31.0)	125 (31.5)	
Other*	20	16 (2.5)	4 (1.0)	
*Morphology* (*n* = 1048, 100%)				0.472
Ductal,	808	509 (78.2)	299 (75.3)	
Lobular/mixed	184	108 (16.6)	76 (19.1)	
Mucinous/tubular/medullary	38	25 (3.8)	13 (3.3)	
Other	18	9 (1.4)	9 (2.3)	
*TNM stadium* (*n* = 1048, 100%)				0.036•
1	172	113 (17.4)	59 (14.9)	
1A	357	241 (37.0)	116 (29.2)	
1B	29	19 (2.9)	10 (2.5)	
2A	248	150 (23.0)	98 (24.7)	
2B	133	69 (10.6)	64 (16.1)	
3A	63	32 (4.9)	31 (7.8)	
3B	11	5 (0.8)	6 (1.5)	
3C	30	19 (2.9)	11 (2.8)	
4	5	3 (0.5)	2 (0.5)	
*Estrogen receptor* (*n* = 1048, 100%)				0.525
Positive	886	545 (83.7)	341 (85.9)	
Negative	153	101 (15.5)	52 (13.1)	
Unknown	9	5 (0.8)	4 (1.0)	
*Progesterone receptor* (*n* = 1048, 100%)				0.206
Positive	710	434 (66.7)	276 (69.5)	
Negative	321	209 (32.1)	112 (28.2)	
Unknown	17	8 (1.2)	9 (2.3)	
Her2Neu receptor (*n* = 1048, 100%)				0.894
Positive	124	75 (11.5)	49 (12.3)	
Negative	893	556 (85.4)	337 (84.9)	
Unknown	31	20 (3.1)	11 (2.8)	
*Molecular subtypes* (*n* = 1048, 100%)				0.552
Triple-negative	109	74 (11.4)	35 (8.8)	
Luminal A	777	478 (73.4)	299 (75.3)	
Luminal B	88	52 (8.0)	36 (9.1)	
HER2-enriched	33	23 (3.5)	10 (2.5)	
Unknown	41	24 (3.7)	17 (4.3)	

*SD* standard deviation, *TNM* tumor–node–metastasis, *Her2Neu* human epidermal growth receptor.

^*^“Other” is a combined category consisting of the following parts: nipple/areola, axillary spur, and mamma not specified; “•” indicates statistical significance. Note: variables as number (percentage)

**Table 3:** Treatment characteristics

Treatment characteristics	Number of patients (*n*)	No pain (*n* = 651, 62.1%)	Persistent pain (*n* = 397, 37.9%)	*p*-Value
*Time since primary surgery, years* (*SD*) (*n* = 1048, 100%)		7.23 (±3.48)	7.26 (±3.39)	0.913
0–5 years	331	211 (32.4)	120 (30.2)	
5–10 years	462	276 (42.4)	186 (46.9)	
10–15 years	255	164 (25.2)	91 (22.9)	
*Type of definitive surgery* (*n* = 1048, 100%)				0.941
Breast-conserving surgery	698	433 (66.5)	265 (66.8)	
Mastectomy	266	167 (25.7)	99 (24.9)	
Mastectomy combined with reconstructive surgery	84	51 (7.8)	33 (8.3)	
*Type of axillary surgery* (*n* = 1048, 100%)				0.000•
SNB alone	744	486 (74.7)	258 (65.0)	
SNB + ALND	122	54 (8.3)	68 (17.1)	
ALND alone	145	86 (13.2)	59 (14.9)	
None	37	25 (3.8)	12 (3.0)	
*Chemotherapy* (*n* = 1048, 100%)				0.000•
Yes	473	261 (40.1)	212 (53.4)	
No	575	390 (59.9)	185 (46.6)	
*Timing of chemotherapy* (*n* = 473, 45.1%)				0.590**
Neoadjuvant	162	92 (35.2)	70 (33)	
Adjuvant	310	168 (64.4)	142 (67)	
Combined	1	1 (0.4)	0 (0)	
*Type of chemotherapy* (*n* = 473, 45.1%)				0.228
TAC	297	162 (62.1)	135 (63.7)	
FEC	119	67 (25.7)	52 (24.5)	
TAC + FEC	30	13 (5.0)	17 (8.0)	
Other/unknown	27	19 (7.3)	8 (3.8)	
*Radiotherapy* (*n* = 1048, 100%)				0.122
Yes	821	500 (76.8)	321 (80.9)	
No	227	151 (23.2)	76 (19.1)	
Localization local radiotherapy (*n* = 821, 78.3%)				0.055***
Mamma	697	433 (86.6)	264 (82.2)	
Chest wall	121	64 (12.8)	57 (17.8)	
Only locoregional	3	3 (0.6)	0 (0)	
*Locoregional radiotherapy* (*n* = 821, 78.3%)				0.168
Axilla	80	49 (9.8)	31 (9.7)	
Periclavicular	25	14 (2.8)	11 (3.4)	
Axilla and periclavicular	43	21 (4.2)	22 (6.9)	
None	611	371 (74.2)	240 (74.8)	
Other****	62	45 (9.0)	17 (5.3)	
*Endocrine therapy* (*n* = 1048, 100%)				0.025•
Yes	566	334 (51.3)	232 (58.4)	
No	482	317 (48.7)	165 (41.6)	
Targeted therapy (*n* = 1048, 100%)				0.558
Yes	119	71 (10.9)	48 (12.1)	
No	929	580 (89.1)	349 (87.9)	

*SD* standard deviation, *SNB* sentinel node biopsy, *ALND* axillary lymph node dissection, *TAC* combination of taxotere, adriamycin and cyclophosphamide, *FEC* combination of fluorouracil, epirubicin, and cyclophosphamide.

^**^To create this *p*-value, the patients with combined treatment were excluded from analysis. ***To create this *p*-value, the patients with only locoregional treatment were excluded from analysis.

^****^“Other” is a combined category consisting of the patient with locoregional radiotherapy in the combined regions of “periclavicular + parasternal,” “axilla + periclavicular + parasternal,” “periclavicular + neck,” and “unknown.” “•” indicates statistical significance. Note: variables as number (percentage)

Pain characteristics for all 397 (37.9%) patients with PPBCT are described in Table 4. Of these patients, 50.8% categorized the pain intensity as moderate to severe based on a numerical rating scale. Almost half of the patients (43.2%) noted that the pain was always present but varied in intensity, and 19.1% characterized their pain as neuropathic based on the DN4 questionnaire.

**Table 4:** Pain characteristics

Pain characteristics	Persistent pain (*n* = 397)
*Most painful location*	
Breast	184 (46.4)
Lateral side of thorax	75 (19.0)
Axilla	66 (16.7)
Arm	51 (12.9)
Unknown	19 (4.8)
NRS scale, median (25%; 75%)	5 (3; 6)
Mild (≤ 4)	194 (49.2)
Moderate/severe (> 4)	200 (50.8)
*VRS scale*	
None or mild, *n* (%)	170 (43.4)
Moderate, *n* (%)	172 (43.9)
Severe and very severe, *n* (%)	50 (12.8)
*Duration of pain, years (SD)*	5.6 (±3.8)
*Start of pain*	
Sudden	107 (27.9)
Gradually	276 (72.1)
*Pain located at one spot only*	
Yes	327 (82.8)
No	68 (17.2)
*Radiation of pain*	
Yes	119 (30.4)
No	273 (69.6)
*Shooting pain that changes location*	
Yes	53 (13.6)
No	336 (84.4)
*Description of pain*	
Pain comes in attacks	158 (41.1)
Pain varies in intensity, but always present	166 (43.2)
Pain continuously present	60 (15.6)
*Neuropathic pain*	
Yes (DN4 score ≥ 4)	61 (19.1)
No (DN4 score < 4)	259 (80.9)
Interference with daily life score (0–54), mean (SD)	16.4 (±15.2)
Mild (NRS ≤ 4), mean (SD)	9.6 (±11.8)
Moderate/severe (> 4), mean (SD)	23.0 (±15.4)
*Need for pain medication*	
Yes	123 (31.1)
No	272 (68.9)
*Type of pain medication*	
Paracetamol	48 (44.4)
NSAID	13 (12.0)
Opioids	10 (9.3)
Other*	37 (34.3)

*NRS* numerical rating scale, *VRS* verbal rating scale, *SD* standard deviation, *DN4* Douleur Neuropathique, *NSAID* nonsteroidal anti-inflammatory analgesic.

^*^“Other” is a combined category consisting of various combinations of the above-mentioned analgesic medication with or without additional neuropathic pain medication (pregabalin and/or amitriptyline). Note: variables as number (percentage)

### Risk Factors of PPBCT

Univariate analysis identified eight variables potentially associated with the reporting of PPBCT (Table 5). These were age at diagnosis of 50 years or older, body mass index (BMI) above 40 kg/m^2^, patients having quit smoking, anxiety, depression, axillary lymph node dissection, chemotherapy, and endocrine therapy.

**Table 5:** Univariate and multivariate analysis

Risk factor	Univariate analysis		Multivariate analysis (*n* = 999)	
	OR (95% CI)	*p*-Value	OR (95% CI)	*p*-Value
*Age at diagnosis, years*				
< 40	1		1	
40–50	0.83 (0.46–1.50)	0.543	0.74 (0.40–1.38)	0.343
50–70	0.63 (0.37–1.09)	0.098*	0.64 (0.36–1.16)	0.145
≥ 70	0.34 (0.18–0.64)	0.001*	0.35 (0.17–0.74)	0.006•
*BMI at baseline, kg/m*^2^				
18–25	1		1	
< 18	1.66 (0.41–6.67)	0.478	1.86 (0.39–8.85)	0.433
25–30	1.00 (0.71–1.41)	0.985	1.11 (0.76–1.61)	0.590
30–35	1.09 (0.68–1.75)	0.730	1.05 (0.62–1.76)	0.859
35–40	0.90 (0.35–2.30)	0.817	0.74 (0.26–2.11)	0.574
≥ 40	3.32 (1.11–9.95)	0.032*	4.43 (1.28–15.40)	0.019•
Unknown	0.96 (0.70–1.31)	0.802	1.02 (0.72–1.45)	0.918
*Smoking*				
No	1		1	
Yes	1.27 (0.82–1.97)	0.289	1.06 (0.65–1.72)	0.821
Quit	1.58 (1.17–2.14)	0.003*	1.55 (1.12–2.14)	0.008•
*Anxiety*				
No (HADS < 8)	1		1	
Yes (HADS ≥ 8)	2.83 (2.06–3.88)	0.000*	2.09 (1.44–3.05)	0.000•
Depression				
No (HADS < 8)	1		1	
Yes (HADS ≥ 8)	3.24 (2.18–4.81)	0.000*	2.51 (1.55–4.07)	0.000•
*Type of axillary surgery*				
None	1		1	
SNB alone	1.11 (0.55–2.24)	0.779	0.92 (0.43–1.97)	0.832
ALND alone	1.43 (0.67–3.07)	0.359	1.00 (0.43–2.33)	0.993
SNB + ALND	2.62 (1.21–5.70)	0.015*	1.91 (0.81–4.50)	0.141
*Chemotherapy*				
No	1		1	
Yes	1.71 (1.33–2.20)	0.000*	1.17 (0.83–1.66)	0.374
*Radiotherapy*				
No	1			
Yes	1.28 (0.94–1.74)	0.123		
*Endocrine therapy*				
No	1		1	
Yes	1.33 (1.04–1.72)	0.025*	1.05 (0.78–1.43)	0.740

*BMI* body mass index, *HADS* hospital anxiety and depression scale, *SNB* sentinel node biopsy, *ALND* axillary lymph node dissection.

^*^p-Value < 0.1; “•” indicates statistical significance

Multivariable logistic regression analysis showed that age at diagnosis of 70 years or older (OR 0.35, *p* = 0.006, for age ≥ 70 versus < 40 years) was independently associated with PPBCT and that the risk of PPBCT was lower in this age category. The categories of BMI above 40 kg/m^2^ (OR 4.43, *p* = 0.019 for BMI ≥ 40 versus BMI between 18 and 25 kg/m^2^), having quit smoking (OR 1.55, *p* = 0.008, for those who quit smoking compared with nonsmokers), anxiety (OR 2.09, *p* < 0.001, for HADS ≥ 8 versus < 8) and depression (OR 2.51, *p* < 0.001, for HADS ≥ 8 versus < 8) were also independently associated with a higher risk of PPBCT (Table 5).

## Discussion

To our knowledge, this work represents one of the largest studies to date describing PPBCT, encompassing a thorough examination of potential risk factors, including demographic, treatment, and psychosocial factors. We investigated 1048 women who had undergone surgical therapy for breast cancer with the aim of examining the prevalence of PPBCT and the association between PPBCT and various patient, tumor, and treatment characteristics. Thus, by using data from the Eindhoven Cancer Registry, we were able to obtain results based on a large, unselected, population-based patient population.

Our findings demonstrate a prevalence of PPBCT of 38% in the population studied in this report. Previous studies that investigated the prevalence of PPBCT have reported a prevalence ranging from 20 to 60%.^9–11^ It is likely that part of this wide range in reported prevalence is caused by a lack of consistency in the definition of PPBCT applied in these reports. We defined PPBCT as pain located in the breast, lateral thorax, axilla, and/or medial upper arm, with or without sensory disturbances, persisting for more than 3 months after surgical breast cancer treatment. Some studies have defined PPBCT as “any pain mentioned by patients” lasting for at least 2–6 months after surgical intervention,^8,10^ whereas one study used a cutoff value of 4 on a numerical rating scale 0–10.^11^ This latter study reported a prevalence of 38.3% in a cohort of 261 patients.^11^ Our results corroborate these previous studies concerning the prevalence of PPBCT in surgical patients with breast cancer.

In the patient population studied in this report, 50.8% of women with PPBCT characterize the pain as moderate to severe (NRS > 4). Our findings show that pain is more likely to be constantly present (43.2%) and to have a gradual onset (72.1%). The prevalence of neuropathic pain was 19%. In their 2017 systematic review, Ilhan et al. reported that the prevalence of neuropathic pain among women treated for early-stage breast cancer ranged between 3.1 and 50.0%.^28^ Based on previously reported findings^9,14,15^ and the present results, PPBCT can be characterized as moderate to severe pain (NRS > 4) with a neuropathic pain component. Nevertheless, there still remains a need for detailed physical examination of patients with signs of neuropathic pain because data used in this study were only based on questionnaires without quantitative sensory testing. Extensive physical examination may provide a rational basis for mechanism-based interventions for PPBCT in the future. Additionally, while the DN4 employs an accepted cutoff value of 4 for identifying neuropathic pain, it is crucial to recognize that this threshold was primarily designed and validated for diagnosing neuropathic pain in general, rather than specifically in the context of post-surgical injury. Thus, we recommend considering this aspect in future studies.

As stated before, studies that examined the significance of risk factors associated with PPBCT provided contradicting results. In one of the larger studies, which included 3253 patients with a 2-year follow-up, 47% of the respondents reported pain.^15^ Of note, this previous study showed that younger patients (< 40 years old), patients treated with adjuvant radiotherapy and patients who underwent axillary lymph node dissection had a significantly higher risk of developing PPBCT. Our study also demonstrated that older patients (age > 70 years at diagnosis) experienced significantly less pain in comparison with younger patients. The univariate analyses used in this report established a strong association between various treatments such as type of axillary surgery, chemotherapy, and endocrine therapy and PPBCT, which is consistent with other studies.^6,10,15,23^ However, these associations were no longer statistically significant after adjustment for confounders in a multivariable analysis.

Our findings show a significant association between a BMI above 40 kg/m^2^ and PPBCT, and between psychosocial status, including the variables anxiety and depression, and PPBCT. These findings are in concordance with existing literature.^15,21,22^ However, no hard conclusions can be drawn regarding a causal pathway that underlies these associations. Because our study used a cross-sectional design, and there were no questions regarding BMI included in the distributed patient questionnaires, there was a considerable amount of missing information on BMI in our study. It also has to be noted that the subgroup of patients with BMI > 40, consisted of only 15 patients, explaining the wide 95% confidence interval in the calculated odds ratio. Regarding the association between anxiety, depression, and PPBCT, some evidence is emerging that behavioral interventions directed at these psychological conditions during the peri- and postoperative period may help prevent the onset of chronic postoperative pain.^21^ The identification of these conditions as potential predictors of PPBCT underscores the importance of preoperative screening. Implementing standardized preoperative assessments that include screening for psychosocial factors can aid in identifying patients at high risk of developing PPBCT and facilitate the application to targeted preventive therapies. Large-scale therapeutic interventions, conducted in relevant subpopulations, are required to outline rational strategies for prevention and treatment of PPBCT. Hopefully, the ongoing AMAZONE study,^30^ a multicenter randomized controlled trial investigating the effect of online cognitive behavioral therapy (e-CBT) on the prevalence of PPBCT, will better clarify potentially beneficial treatment strategies.

Surprisingly, radiotherapy did not show an association with the reporting of PPBCT in our univariate analyses. We included this variable on the basis of contradictory evidence in the literature. Specifically, some studies concluded that radiotherapy could be related to the development of PPBCT,^10,23^ whereas another study found no association.^6^ It is generally accepted that women who receive radiotherapy are at risk of late side effects including radiation-induced fibrosis. This is an irreversible condition in which excessive formation of fibrous connective tissue causes structural and functional changes^29^ that are thought to induce neuropathy and neuropathic pain. Further prospective research, taking into account the radiation techniques and doses and the irradiated fields,^31^ is needed to explore these contradictory findings and to establish conclusive evidence regarding the relationship between radiotherapy and PPBCT.

The strengths of this study are that it is based on a large representative cohort that reflects usual breast cancer care in the Netherlands and that it is large enough to provide reasonably precise risk estimates of the prevalence of PPBCT and the strength of the association with patient, tumor, and treatment variables. Our results on the effect of axillary surgery, chemotherapy, and endocrine therapy demonstrate the importance of multivariate analyses to unravel pain risk factors independent of those linked to persistent pain. Many previous studies have been published based on relatively small sample sizes that often focused on only one or two variables.^11,26,27^ The questionnaires used in this study covered not only the severity or intensity of pain but also many other aspects regarding treatment and patient characteristics. Incorporating patient-reported outcome measures (PROMS) into preoperative assessment can provide valuable insights into patients’ experiences and perceptions of pain, thus aiding in individualized treatment planning and monitoring of postoperative outcomes. This approach aligns with the growing emphasis on patient-centered care and personalized medicine.

The principal limitation of this study is its cross-sectional design. We did not follow up patients over time, and thus, we are unable to comment on how the reported pain develops over time. Another limitation is that some of the included patients underwent breast cancer treatment more than 10 years ago. This introduces the potential for bias, as these patients may report pain differently compared with those who were treated more recently. This could be due to several factors, including changing perceptions of pain over time, differences in treatment modalities, and advancements in pain management techniques over the years. Therefore, it is important to consider this variability when interpreting the results, as the experience and reporting of pain may vary among patients who underwent treatment during different time periods. Additionally, the cross-sectional design used in this study does not allow us to draw conclusions regarding causality but can merely describe associations between various variables and persistent pain. Another limitation of this study is the relatively high nonresponder rate of 43.5%, which may introduce potential selection bias. However, it is noteworthy that, despite this limitation, a considerable number of participants, comprising more than half of the total, did actively respond and provide valuable data for analysis.

This large, cross-sectional, retrospective cohort study conducted in a Dutch teaching hospital showed that PPBCT is a frequently observed phenomenon among breast cancer survivors, affecting 37.9% of patients. Anxiety, depression, and a history of smoking are significantly associated with increased reporting of PPBCT, though causation is not implied. Patients aged 70 or older at diagnosis are less susceptible. Treatment type may not impact PPBCT reporting significantly, but further research is needed. Prospective studies are necessary to identify potential intervention targets for PPBCT prevention and treatment. These findings emphasize the importance of understanding PPBCT and developing targeted interventions to improve patient well-being, particularly during preoperative counseling. Proactive pain management strategies can potentially mitigate the burden of PPBCT and enhance the overall quality of care for patients with breast cancer undergoing surgery.
